# Validation of PhageDx™ *Cronobacter* Assay for the Identification of *Cronobacter* Spp. in Powdered Infant Formula: AOAC *Performance Tested Method*^SM^ 051803

**DOI:** 10.1093/jaoacint/qsab038

**Published:** 2021-03-18

**Authors:** Stephen Erickson, Jose Gil, Jessica Stach, Wendy Hahn, Minh M Nguyen

**Affiliations:** 1 Laboratory Corporation of America/MedTox, 402 County Road D West, St. Paul, MN 55112, USA; 2 Laboratory of America Corporation/National Genetics Institute, 2440 Sepulveda Blvd, Suite 235, Los Angeles, CA 90064, USA

## Abstract

**Background:**

The PhageDx^™^*Cronobacter* Assay is based on the infection of *Cronobacter* spp. by specific bacteriophages and expression of a luciferase reporter gene. Results are generated in as little as 18.5 h for powdered infant formula (PIF).

**Objective:**

An AOAC *Performance Tested Methods*^SM^ (PTM) study was conducted to validate the PhageDx *Cronobacter* Assay for the detection of *Cronobacter* in 10, 100, and 300 g milk- and soy-based PIF test portions.

**Method:**

The performance of the PhageDx method was compared to the ISO 22964:2006/2017 Microbiology of the Food Chain—Horizontal Method for the Detection of *Cronobacter* spp. and the U.S. Food and Drug Administration (FDA) *Bacteriological Analytical Manual* (BAM) Chapter 29 *Cronobacter*: 2012. Inclusivity/exclusivity, product consistency and stability, and robustness testing also were conducted.

**Results:**

There was no significant difference between the 10, 100, or 300 g test portions for the milk and soy PIF matrixes between the PhageDx *Cronobacter* Assay, the ISO 22964:2006/2017, and the FDA BAM Chapter 29 *Cronobacter*: 2012 methods. The reporter bacteriophages were specific for *Cronobacter* and infected 75 strains in inclusivity testing. They did not infect 35 non-*Cronobacter* bacteria in exclusivity testing. Robustness testing showed that the method performed well with specific deviations from the standard protocol. Consistency and stability testing demonstrated that the recombinant phage gave consistent results across three production lots and was stable when stored under appropriate conditions for at least 3 months.

**Conclusions:**

Work in the submitting and independent laboratories demonstrated that the PhageDx *Cronobacter* Assay meets the qualifications for PTM status.

**Highlights:**

The PhageDx *Cronobacter* Assay is a rapid, simple, and specific test that has shown equivalence to both the FDA BAM and ISO reference methods for detecting *Cronobacter* spp. in PIF.

## General Information

*Cronobacter*, formerly classified under *Enterobacter*, are bacteria that are resistant to desiccation, heat, and ultraviolet radiation. In infants, particularly neonates, *Cronobacter* can cause sepsis or severe meningitis resulting in possible long-term neurological issues. It is estimated that the rate of infection for low birth weight infants, who are particularly susceptible, is 8.7 per 100 000 and the mortality rate for infants from *Cronobacter* meningitis can be as high as 40% (5). Nearly all cases of infant *Cronobacter* infections have been associated with the consumption of contaminated powdered infant formula (PIF) (1). As a result, the World Health Organization and the U.S. Food and Drug Administration (FDA) deemed *Cronobacter* a health hazard to neonates that consume PIF contaminated with *Cronobacter,* requiring end product testing for *Cronobacter* (*n* = 30, *c* = 0, 10 g sample) as a compliance requirement before placing PIF on either the U.S. or EU market (6, 7).

## Principle

The PhageDx™ *Cronobacter* Assay is based on the infection of *Cronobacter* spp. by bacteriophages and replication of the infecting bacteriophages within their specific hosts. Bacteriophages demonstrate a high specificity for their bacterial host and are capable of replicating within their host quickly to high numbers. The recombinant phages used in the PhageDx *Cronobacter* Assay also express a luciferase reporter during replication. The presence of *Cronobacter* spp*.* is determined by incubating the lysate with the appropriate luciferase substrate and detecting emitted light in a luminometer. An absence of detected light indicates that no *Cronobacter* are present in that sample. An advantage of this system is that only viable bacteria are detected as bacteriophage only replicate in living cells.

## Scope of Method

- *Target organism*.—*Cronobacter* spp.
- *Matrix*.—PIF (milk-based), PIF (soy-based).
- *Summary of validated performance claims*.—Performance equivalent to that of the U.S. FDA **Bacteriological Analytical Manual** (BAM) Chapter 29 *Cronobacter* (1) and ISO 22964:2006 or ISO 22964:2017 Microbiology of the Food Chain—Horizontal Method for the Detection of *Cronobacter* spp. (2, 3).

## Definitions

- *Probability of detection (POD).*—The proportion of positive analytical outcomes for a qualitative method for a given matrix at a given analyte level or concentration. *POD* is concentration dependent. Several *POD* measures can be calculated: *POD_R_* (reference method *POD*), *POD_C_* (confirmed candidate method *POD*), *POD_CP_* (candidate method presumptive result *POD*), and *POD_CC_* (candidate method confirmation result *POD*).
- *Difference of probabilities of detection (dPOD).*—Difference of probabilities of detection is the difference between any two *POD* values. If the confidence interval of a *dPOD* does not contain zero, then the difference is statistically significant at the 5% level (4).

## Materials and Methods

### Test Kit Information

- *Kit name*.—PhageDx *Cronobacter* Assay.
- *Cat.* No.—5008.
- *Ordering information*.—Not applicable. For internal use at Laboratory Corporation of America only.

### Test Kit Components

- *PhageDx Cronobacter recombinant phage.*—Part. No. 3101, 12 tubes containing 100 µL phage solution.
- *Lysis buffer.*—Part. No. 3002, 12 tubes containing 100 µL lysis buffer.
- *Assay buffer.*—Part. No. 3003, 12 tubes containing 500 µL assay buffer.
- *Luciferase substrate.*—Part. No. 3004, 12 tubes containing 10 µL luciferase substrate.
- *96-well break-apart plate.*—Part. No. 3103, one pouch containing break-apart plate (8 wells × 12 strips).
- *One package insert.*—Part. No. 3102.

### Additional Supplies and Reagents

- *Sample bags.*—Recommended sample bags: Fisher Scientific, Cat. No. 14-955-187 (10 g); Fisher Scientific Cat. No. 01-812 (100 g); Fisher Scientific, Cat. No. 14-209-300 (300 g).
- *Microfuge tubes (1.5 mL).*
- *Sample bag and tube racks.*
- *Buffered peptone water (BPW).*—Thermo Fisher Scientific, Cat. No. CM0509.
- *Sample pipettor (2–5 mL).*
- *Sterile, filtered pipet (2–5 mL).*
- *Adjustable single channel pipettor (10 µL–1 mL) and appropriate sterile tip*s.
- *Appropriate personal protective equipment.*—*See* (Ref. 8).
- *Thermo Scientific Oxoid^™^ Brilliance^™^ Cronobacter sakazakii agar.*—Thermo Fisher Scientific Cat. No. CM1055B.

### Apparatus

- *Homogenizer.*—Seward Stomacher^®^ 400/3500 or similar.
- *Air incubators capable of 37 ± 1°C.*
- *Promega GloMax^®^ 96 or Navigator luminometer.*
- *Personal computer for luminometer control and data analysis.*

### Safety Precautions

- The PhageDx *Cronobacter* Assay involves the enrichment of samples which may contain human pathogenic *Cronobacter* and have the potential for contamination with subsequent handling of those samples. This method should be conducted by properly trained laboratory personnel in a suitable microbiology laboratory in accordance with “Biosafety in Microbiological and Biomedical Laboratories” (8). Care should be taken when handling the sample and reagents while performing the method.
- Materials and reagents provided in the PhageDx *Cronobacter* Assay are not considered hazardous if used according to the assay method. Please review the Material Safety Data Sheet prior to performing the assay.
- Follow all relevant guidelines and laboratory protocols while performing the assay and manufacturer’s equipment instructions.

### General Preparation

- Prepare BPW media according to manufacturer’s instructions.
- Before using the reagents, flick or spin the tube to collect all of the solution at the bottom of the tube.
- Due to the short enrichment times, it is vital to maintain the temperature of the sample and BPW media used in the incubation.
- Before adding the pre-warmed BPW to the sample, confirm that the media and incubator are warmed to 37 ± 1°C.
- Do not allow the pre-warmed media to cool before adding to the sample.
- Maintain the media at 37 ± 1°C in an incubator or water bath if preparing multiple samples.

### Sample Preparation

- Weigh 10, 100, or 300 g PIF and place into a sample bag.
- Add 90 mL (10 g test portion), 300 mL (100 g test portion), or 900 mL (300 g test portion) pre-warmed (37 ± 1°C) BPW to the sample.
- Homogenize sample in a Stomacher 400 or Stomacher 3500, depending on sample size, at the highest setting for 120 s (or equivalent homogenizer and setting).
- Loosely close the sample bag and place in a static air incubator at 37 ± 1°C for 16–18 h using a sample rack to keep the bags separate and allow for heat transfer.
- Remove the enriched samples from the incubator and mix thoroughly by hand for at least 30 s or stomach to ensure complete mixing.

*Note:* Sample must be thoroughly mixed so the analyte is distributed evenly throughout the entire sample. We recommend vigorous shaking and massaging for at least 30 s. Immediately proceed to the next step after mixing is complete. If sample sits for 15 min or longer, mix sample again before proceeding to the next step.

**(f)** Using a pipettor with a sterile tip, transfer 1 mL sample to a sterile 1.5 mL microfuge tube.
**(g)** Mix contents in microfuge tube and dilute sample 1:10 in BPW (100 µL sample in 900 µL BPW).
**(h)** Using a single channel pipettor and clean tip for each sample, transfer 150 µL diluted sample to 96-well plate.
**(i)** Using a single channel pipettor and clean tip for each sample, add 10 µL phage solution to the sample and gently mix by pipetting up and down.
**(j)** Cover plate with plate sealing tape and place the sample in the 37 ± 1°C incubator for 2 h.
**(k)** Remove one tube containing the lysis buffer, assay buffer, and substrate for each eight-well strip used and thaw to room temperature. Flick or spin the tubes to collect all of the solution at the bottom of the tubes.
**(l)** Prepare the luciferase substrate working solution by transferring the entire contents of assay buffer (0.5 mL) to the substrate tube (10 µL) and mix well.

*Note:* Use within 1 h of preparation.

**(m)** Using a clean tip for each sample, add 10 µL lysis buffer and mix thoroughly by gently pipetting up and down being careful not to introduce bubbles.
**(n)** Add 50 µL 1:50 luciferase substrate working solution to each well using a single channel pipettor and clean tip for each sample. Mix thoroughly by gently pipetting up and down, being careful not to introduce bubbles.
**(o)** Once all of the samples have received the substrate, place the sample plate in the luminometer, close the lid, and initiate the read program.

### Interpretation and Test Result Report

- The luminometer program will display the results on the screen as relative light unit (RLU) values corresponding to the well positions of the break-away plate.
- Samples positive for *Cronobacter* spp. will have a reading value of 500 RLU or greater. Negative samples will be less than 500 RLU. Note samples that are positive.
- Once all of the samples have been run and analyzed, remove the plate from the luminometer and follow the manufacturer’s instructions for cleaning the instrument and shut down.

### Confirmation

- Confirmation of *Cronobacter* spp. can be performed by streaking 24 h enriched cultures onto Oxoid Brilliance *Cronobacter sakazakii* Agar. To prepare for the confirmation, allow the samples to continue enriching for a total of 24 h (or for an additional 6–8 h) at 37 ± 1°C. Remove 50 µL of the overnight culture and streak onto Oxoid Brilliance *Cronobacter sakazakii* agar and incubate plates for 24 h at 37 ± 1°C.
- Plates with colonies that appear blue-green and grow well are positive.
- Alternative confirmation methods are described in ISO 22964:2017 Microbiology of the Food Chain—Horizontal Method for the Detection of *Cronobacter* spp. (3) and U.S. FDA BAM Chapter 29 *Cronobacter*.

## Validation Study

This validation study was conducted under the AOAC Research Institute (RI) *Performance Test Method(s)*^SM^ program and the *AOAC INTERNATIONAL Methods Committee Guidelines for Validation of Microbiological Methods for Food and Environmental Surfaces, Appendix J* (9). Method developer studies were conducted in the laboratories of Laboratory Corporation of America Holdings, and included the inclusivity/exclusivity study, matrix studies for all claim matrixes, product consistency and stability studies, and robustness testing. The independent laboratory study was conducted by Q Laboratories, Inc., and included a matrix study for milk-based PIF.

### Method Developer Studies

####  

##### Inclusivity and exclusivity

Inclusivity strains (*Cronobacter*) were obtained from academic, governmental, and commercially available sources (Table 1). Each strain was grown overnight in tryptic soya broth (TSB) media at 37 ± 1°C until stationary phase. Cells were diluted to 100 CFU in 0.1 mL and mixed with recombinant phage for 2 h at 37 ± 1°C. Following infection, samples were mixed with lysis buffer and luciferase substrate working solution and then read in a luminometer. Samples with RLU values greater than 150 were considered positive. Exclusivity strains were also obtained from commercially available sources and were grown to stationary phase overnight. Assays with exclusivity strains were done as with inclusivity strains except overnight cultures were assayed directly (Table 2).

##### Product Consistency (Lot-to-lot) and Stability

Three separate production lots of PhageDx *Cronobacter* recombinant phage (made on 2/10/2017, 4/28/2017, and 5/5/2017) were prepared according to written manufacturing documents and tested according to quality control procedures. Quality control procedures verified that each lot, when diluted to working concentration, had the similar titer, background, and level of detection (LOD). Recombinant phage lots were aged between 1 and 3 months when assayed for stability.

**Table 1.** Inclusivity list: *Cronobacter*

No.	*Cronobacter* strain	Source	Origin	PhageDx result
1	*Cronobacter sakazakii*	ATCC BAA-894^a^	Food, PIF	Positive
2	*Cronobacter sakazakii*	ATCC12868	Unknown^b^	Positive
3	*Cronobacter sakazakii*	ATCC29004	Unknown	Positive
4	*Cronobacter sakazakii*	ATCC 29544	Clinical	Positive
5	*Cronobacter sakazakii*	FDA E54963-71^c^	Clinical	Positive
6	*Cronobacter sakazakii*	FDA 255N	Clinical	Positive
7	*Cronobacter sakazakii*	FDA CQ31	PIF environment	Positive
8	*Cronobacter sakazakii*	FDA CQ126	PIF environment	Positive
9	*Cronobacter sakazakii*	FDA E788	Clinical	Positive
10	*Cronobacter sakazakii*	FDA CQ123	PIF environment	Positive
11	*Cronobacter sakazakii*	FDA CQ92	PIF environment	Positive
12	*Cronobacter sakazakii*	FDA 2/4/2011	PIF	Positive
13	*Cronobacter sakazakii*	FDA E.sak713	Food, PIF	Positive
14	*Cronobacter sakazakii*	FDA LR834	Environment dairy	Positive
15	*Cronobacter sakazakii*	FDA 2193–02	Clinical	Positive
16	*Cronobacter sakazakii*	FDA LR835	Environment dairy	Positive
17	*Cronobacter sakazakii*	FDA ES9369-75	Clinical	Positive
18	*Cronobacter sakazakii*	FDA ES626	Food, rice flour	Positive
19	*Cronobacter sakazakii*	FDA 708	Clinical	Positive
20	*Cronobacter sakazakii*	FDA ES1059-71	Clinical	Positive
21	*Cronobacter sakazakii*	FDA ES718	Clinical	Positive
22	*Cronobacter sakazakii*	FDA ES717	Food, PIF	Positive
23	*Cronobacter sakazakii*	FDA 2154	Clinical	Positive
24	*Cronobacter sakazakii*	FDA 607A	Clinical	Positive
25	*Cronobacter sakazakii*	FDA 2148	Clinical	Positive
26	*Cronobacter sakazakii*	FDA 2150	Clinical	Positive
27	*Cronobacter sakazakii*	FDA GK792.3	PIF environment	Positive
28	*Cronobacter sakazakii*	FDA GK799	PIF environment	Positive
29	*Cronobacter sakazakii*	FDA GK794	PIF environment	Positive
30	*Cronobacter sakazakii*	FDA GK800	PIF environment	Positive
31	*Cronobacter sakazakii*	FDA GK801.1	PIF environment	Positive
32	*Cronobacter sakazakii*	FDA GK797	PIF environment	Positive
33	*Cronobacter sakazakii*	FDA GK952	PIF environment	Positive
34	*Cronobacter sakazakii*	FDA LR702	Food, PIF	Positive
35	*Cronobacter sakazakii*	FDA LR703	Food, hi PDI^g^ flour	Positive
36	*Cronobacter sakazakii*	FDA LR704	Food, hi PDI flour	Positive
37	*Cronobacter sakazakii*	FDA LR705	Food, organic soy powder	Positive
38	*Cronobacter sakazakii*	FSL F6-0023^d^	Clinical	Positive
39	*Cronobacter sakazakii*	FSL F6-024	Infant formula	Positive
40	*Cronobacter sakazakii*	FSL F6-025	Enviromental	Positive
41	*Cronobacter sakazakii*	FSL F6-027	Enviromental	Positive
42	*Cronobacter sakazakii*	FSL F6-028	Clinical	Positive
43	*Cronobacter sakazakii*	FSL F6-0029	Clinical	Positive
44	*Cronobacter sakazakii*	FSL F6-0034	Clinical	Positive
45	*Cronobacter sakazakii*	FSL F6-035	Clinical	Positive
46	*Cronobacter sakazakii*	FSL F6-0036	Enviromental	Positive
47	*Cronobacter sakazakii*	FSL F6-037	Enviromental	Positive
48	*Cronobacter sakazakii*	FSL F6-0038	Enviromental	Positive
49	*Cronobacter sakazakii*	FSL F6-039	Enviromental	Positive
50	*Cronobacter sakazakii*	FSL F6-0040	Enviromental	Positive
51	*Cronobacter sakazakii*	FSL F6-041	Enviromental	Positive
52	*Cronobacter sakazakii*	FSL F6-042	Infant formula	Positive
53	*Cronobacter sakazakii*	FSL F6-0043	Clinical	Positive
54	*Cronobacter sakazakii*	FSL F6-044	Food	Positive
55	*Cronobacter sakazakii*	FSL F6-045	Food	Positive
56	*Cronobacter sakazakii*	FSL F6-046	Infant formula	Positive
57	*Cronobacter sakazakii*	FSL F6-047	Infant formula	Positive
58	*Cronobacter sakazakii*	FSL F6-048	Infant formula	Positive
59	*Cronobacter sakazakii*	FSL F6-0049	Clinical	Positive
60	*Cronobacter sakazakii*	FSL F6-050	Clinical	Positive
61	*Cronobacter muytjensii*	ATCC 51329	Unknown	Positive
62	*Cronobacter malonaticus*	FDA C1825	Clinical	Positive
63	*Cronobacter malonaticus*	FDA ES686	Food, PIF ingredient	Positive
64	*Cronobacter malonaticus*	FDA ES1895-73	Clinical	Positive
65	*Cronobacter malonaticus*	FDA ES0939A-75	Clinical	Positive
66	*Cronobacter malonaticus*	FSL F6-030	Infant formula	Positive
67	*Cronobacter malonaticus*	FSL F6-0052	Clinical	Positive
68	*Cronobacter malonaticus*	E265^c^	Unknown	Positive
69	*Cronobacter muytjensii*	FSL F6-031	Infant formula	Positive
70	*Cronobacter turicensis*	FDA Z3032	Clinical	Positive
71	*Cronobacter dublinensis*	FDA 5960–70	Clinical	Positive
72	*Cronobacter malonaticus*	CDC 3523–75^e^	Unknown	Positive
73	*Cronobacter dublinensis*	E464^c^	Unknown	Positive
74	*Cronobacter dublinensis*	E515^c^	Unknown	Positive
75	*Cronobacter genomospecies*	NCTC 9529^f^	Unknown	Positive

a ATCC = American Type Culture Collection, Manassas, VA.

b Unknown = No information is available on the origin of the strain.

c Obtained from FDA = U.S. Food and Drug Administration, Center for Food Safety and Applied Nutrition, College Park, ND.

d FSL = Cornell Food Safety Laboratory, Cornell University, Ithaca, NY.

e CDC = Centers for Disease Control and Prevention, Atlanta, GA.

f NCTC = National Collection of Type Cultures, Porton Down, Salisbury, UK.

g PDI = Protein dispersiblity index.

**Table 2.** Exclusivity list

No.	Strain	Source	Origin	PhageDx result
1	*Hafnia alveii*	ATCC 13337^a^	Unknown^b^	Negative
2	*Shigella flexneri*	ATCC 12022	Unknown	Negative
3	*Proteus mirabilis*	ATCC 43071	Clinical, toe	Negative
4	*Edwardsiella tarda*	ATCC 15947	Stool	Negative
5	*Escherichia hermanni*	ATCC 33650	Clinical, toe	Negative
6	*Staphylococcus aureus*	ATCC 27660	Unknown	Negative
7	*Staphylococcus aureus*	ATCC 6538	Human lesion	Negative
8	*Staphylococcus aureus*	ATCC 25923	Clinical	Negative
9	*Enterobacter cloacae*, subsp. *cloacae*	ATCC 13047	Spinal fluid	Positive
10	*Serratia marcescens*	ATCC 13880	Pond water	Negative
11	*Acinetobacter calcoaceticus*	ATCC 23055	Unknown	Negative
12	*Morganella morganii:* subsp*. Maorganii* M11	ATCC 25830	Clinical	Negative
13	*Pseudomonas aeruginosa;* strain Boston 41401	ATCC 27853	Blood culture	Negative
14	*Proteus vulgaris*	ATCC 33420	Clinical	Negative
15	*Enterococcus faecalis*	ATCC 29212	Urine	Negative
16	*Enterobacter aerogenes*	ATCC 13048	Sputum	Positive
17	*Staphylococcus epidermidis*	ATCC 14990	Nose	Negative
18	*Staphylococcus aureus*	ATCC 29213	Wound	Negative
19	*Citrobacter freundii*	ATCC 8090	Unknown	Negative
20	*Shigella sonnei*	ATCC 9290	Unknown	Negative
21	*Klebsiella pneumoniae*	ATCC 4352	Cow’s milk	Negative
22	*Salmonella enterica, *serovar* Choleraesuis*	ATCC 12011	Unknown	Negative
23	*Escherichia fergusonii*	ATCC 35469	Human feces	Negative
24	*Yersinia enterocolitica*	ATCC 23715	Human blood	Negative
25	*Escherichia coli*	ATCC 13706	Unknown	Negative
26	*Escherichia coli*	ATCC 9637	Unknown	Negative
27	*Escherichia coli*	ATCC 4157	Unknown	Negative
28	*Escherichia coli*	ATCC 51813	Food	Negative
29	*Escherichia coli*	ATCC 35421	Unknown	Negative
30	*Escherichia coli*	ATCC 8739	Feces	Negative
31	*Escherichia coli*	ATCC 35218	Canine	Negative
32	*Escherichia coli*	ATCC 11775	Urine	Negative
33	*Escherichia coli*	ATCC 25922	Clinical	Negative
34	*Enterobacter asburiae*	FSL F6-0026^c^	Environmental	Positive
35	*Salmonella enterica, *serovar* Anatum*	ATCC9270	Pork liver	Negative
36	*Citrobacter koseri*	ATCC 25408	Throat	Negative
37	*Citrobacter braakii*	ATCC 51113	Snake	Negative
38	*Pluralibacter gergoviae*	ATCC 33028	Urine	Negative

a ATCC = American Type Culture Collection, Manassas, VA.

b Unknown = No information is available on the origin of the strain.

c FSL = Cornell Food Safety Laboratory, Cornell University, Ithaca, NY.

Consistency and stability were done according to AOAC guidance, where a sample was inoculated with *Cronobacter malonaticus* ES686, a strain isolated from an ingredient in PIF, to give fractional positives. Ten replicates were run in the PhageDx *Cronobacter* Assay, and the RLU values analyzed. A set of stability studies was also conducted using the non-target bacterium *Citrobacter koseri* (ATCC 25408). Overnight cultures of *C. koseri* were used directly in the assay. Results are shown in Table 3.

**Table 3.** Stability and consistency (lot-to-lot) of PhageDx *Cronobacter* recombinant phage—POD comparison

Phage lot No.	Lot age, months	*n* ^a^	x^b^	POD_A_^c^	95% CI	Phage lot No.	Lot age, months	*n*	x	POD_B_^d^	95% CI	dPOD_AB_^e^	95% CI^f^
*Cronobacter malonaticus* (target)													
0217^g^	2	10	6	0.6	0.31, 0.83	0517^i^	1	10	6	0.6	0.31, 0.83	0.0	−0.37, 0.37
0417^h^	3	10	5	0.5	0.24, 0.76	0517	1	10	6	0.6	0.31, 0.83	−0.10	−0.45, 0.29
0417	3	10	5	0.5	0.24, 0.76	0217	2	10	6	0.6	0.31, 0.83	−0.10	−0.45, 0.29
*Citrobacter koseri* (non-target)													
0217	2	10	0	0.0	0.0, 0.28	0517	1	10	0	0.0	0.0, 0.28	0.0	−0.28, 0.28
0417	3	10	0	0.0	0.0, 0.28	0517	1	10	0	0.0	0.0, 0.28	0.0	−0.28, 0.28
0417	3	10	0	0.0	0.0, 0.28	0217	2	10	0	0.0	0.0, 0.28	0.0	−0.28, 0.28

a *n* = Number of test portions.

b x = Number of positive test portions.

c POD_A_ = Positive outcomes divided by the total number of trials first member of pair.

d POD_B_ = Positive outcomes divided by the total number of trials second member of pair.

e dPOD_AB_ = Difference in POD between the paired comparison.

f 95% CI = If the confidence interval of a dPOD does not contain zero, then the difference is statistically significant at the 5% level.

g Lot 0217 was produced 2/10/2017.

h Lot 0417 was produced 4/28/17.

i Lot 0517 was produced 5/5/2017.

##### Robustness

Three parameters were varied to demonstrate assay robustness: enrichment time (14 and 24 h), recombinant phage concentration (±20%), and luciferase substrate amount (±10%). Briefly, 10 g milk-based PIF samples were left unspiked or spiked with 0.2–2 CFU/10 g *Cronobacter muytjensii* FSL-F6-031 dried in PIF and stored at room temperature (20–25°C) for 2–4 weeks. The PhageDx *Cronobacter* Assay protocol was followed with the variations in enrichment time, recombinant phage concentration, and substrate amount as indicated in Table 4. Samples with RLU values greater than 500 were considered positive. Samples were confirmed by allowing samples to enrich for a total of 24 ± 2 h and then plating on Oxoid Brilliance *Cronobacter sakazakii* agar*.* Plates were incubated at 37 ± 1°C for an additional 24 ± 2 h. The presence of blue-green colonies that grew well indicated positive samples. A summary of the testing is presented in Table 4.

**Table 4.** Robustness study: impact of varying enrichment time, phage concentration, and luciferase substrate concentration on PhageDx *Cronobacter* Assay results—POD comparison

Test condition^a^	Test parameters			*n* ^b^	Test condition results				Nominal condition results^e^			dPOD_TN_^g^	95% CI^h^
	Enrichment time, h	Volume phage, µL	Volume substrate		x^c^	POD_T_^d^	95% CI		x	POD_N_^f^	95% CI		
Milk-based PIF—spiked with *Cronobacter muytjensii* (target)													

1	14	8	45	10	5	0.5	0.24, 0.76		5	0.5	0.24, 0.76	0.0	−0.37, 0.37
2	14	8	55	10	5	0.5	0.24, 0.76		5	0.5	0.24, 0.76	0.0	−0.37, 0.37
3	14	12	45	10	5	0.5	0.24, 0.76		5	0.5	0.24, 0.76	0.0	−0.37, 0.37
4	14	12	55	10	5	0.5	0.24, 0.76		5	0.5	0.24, 0.76	0.0	−0.37, 0.37
5	24	8	45	10	5	0.5	0.24, 0.76		5	0.5	0.24, 0.76	0.0	−0.37, 0.37
6	24	8	55	10	5	0.5	0.24, 0.76		5	0.5	0.24, 0.76	0.0	−0.37, 0.37
7	24	12	45	10	5	0.5	0.24, 0.76		5	0.5	0.24, 0.76	0.0	−0.37, 0.37
8	24	12	55	10	5	0.5	0.24, 0.76		5	0.5	0.24, 0.76	0.0	−0.37, 0.37

Milk-based PIF—unspiked (non-target)													

1	14	8	45	10	0	0.0	0.00, 0.28		0	0.0	0.00, 0.28	0.0	−0.25, 0.25
2	14	8	55	10	0	0.0	0.00, 0.28		0	0.0	0.00, 0.28	0.0	−0.25, 0.25
3	14	12	45	10	0	0.0	0.00, 0.28		0	0.0	0.00, 0.28	0.0	−0.25, 0.25
4	14	12	55	10	0	0.0	0.00, 0.28		0	0.0	0.00, 0.28	0.0	−0.25, 0.25
5	24	8	45	10	0	0.0	0.00, 0.28		0	0.0	0.00, 0.28	0.0	−0.25, 0.25
6	24	8	55	10	0	0.0	0.00, 0.28		0	0.0	0.00, 0.28	0.0	−0.25, 0.25
7	24	12	45	10	0	0.0	0.00, 0.28		0	0.0	0.00, 0.28	0.0	−0.25, 0.25
8	24	12	55	10	0	0.0	0.00, 0.28		0	0.0	0.00, 0.28	0.0	−0.25, 0.25

a Each test condition is being compared to the nominal test condition. *Note*: Test conditions 1–4 (14 h enrichment) and test conditions 5–8 (24 h enrichment) were compared to the nominal condition in different experiments.

b *n* = Number of test portions per condition.

c x = Number of positive test portions per condition.

d POD_T_ = Positive outcomes divided by the total number of trials per condition.

e Nominal condition = 16 h Enrichment, 10 µL phage, 50 µL luciferase working solution.

f POD_N_ = Positive outcomes divided by the total number of trials per nominal condition.

g dPOD_TN_ = Difference in POD between the test condition and nominal condition.

h 95% CI = If the confidence interval of a dPOD does not contain zero, then the difference is statistically significant at the 5% level.

##### Matrix Study

The matrix study compared the PhageDx *Cronobacter* (10 g test portions) to ISO 22964:2006 (10 g test portions) and the PhageDx *Cronobacter* (100 and 300 g test portions) to FDA BAM Chapter 29 *Cronobacter*: 2012 (100 g test portions). The PhageDx *Cronobacter* 10 g portions were compared to ISO 22964:2006 using a paired study design. The PhageDx *Cronobacter* 100 and 300 g portions were compared to the FDA BAM Chapter 29 100 g portions using an unpaired study design. For each matrix and each comparison, the study included five replicate test portions of uninoculated matrix (0 CFU/test portion), 20 replicate test portions at a low level to yield fractionally positive results (0.2–2 CFU/test portion), and five replicate test portions at a high level to yield consistently positive results (2–10 CFU/test portion).

Both milk-based and soy-based PIF were purchased from local retail stores and prescreened for natural contamination using the ISO 22964:2006 method. To prepare the inoculum, *Cronobacter* was grown in TSB for 18–24 h at 37 ± 1°C. The culture was diluted in BPW, reconstituted in PIF, and placed into a speed vacuum for 4–8 h until the sample was completely dried. After desiccation, the dried inoculum was diluted into the PIF matrix used in each study to obtain a low level, expected to yield fractional positive results, and a high level, expected to yield all positive results, and allowed to sit for 2–4 weeks at room temperature (20–25°C) to allow for equilibration in the matrix. A bulk lot of the matrix was inoculated with the diluted inoculum prior to testing.

On the day of analysis, total aerobic count was determined according to FDA BAM Chapter 3 (10) and the level of *Cronobacter* in low level and high level inoculum was determined by most probable number (MPN) analysis. For the paired samples, MPN analysis was determined using the ISO 22694:2006 method. For low level inoculum, five test portions of 25 g, five test portions of 4 g, and 20 test portions of 10 g from the matrix study were analyzed. For the high level inoculum, five test portions of 10 g from the matrix study, five test portions of 4 g, and five test portions of 1.5 g were analyzed.

For the unpaired samples, MPN analysis was determined using the FDA BAM Chapter 29 method. For low level inoculum, five test portions of 200 g, five test portions of 50 g, and 20 test portions of 100 g from the matrix study were analyzed. For the high level inoculum, five test portions of 100 g from the matrix study, five test portions of 50 g, and five test portions of 25 g were analyzed. The number of positives was used to calculate the MPN using the Least Cost Formulations Most Probable Number (LCF MPN) calculator provided by AOAC RI (11).

##### PhageDx *Cronobacter* Assay

Test portions were processed according to directions for use. Briefly, 90 mL (10 g test portion), 300 mL (100 g test portion), or 900 mL (300 g test portion) of pre-warmed BPW (37 ± 1°C) was added to PIF test portions. Samples were homogenized and enriched at 37 ± 1°C for 16–18 h. Enriched samples were mixed thoroughly before taking aliquots for analysis. Samples were diluted 1:10 (100 µL sample: 900 µL BPW) in pre-warmed BPW (37 ± 1°C) and 150 µL diluted sample was transferred to a 96-well plate. Samples were then infected with recombinant phage for 2 h at 37 ± 1°C. Lysis buffer and luciferase substrate working solution were added to the samples. Samples were then read on a luminometer. Readings of ≥500 RLU were considered positive. To confirm per PhageDx *Cronobacter* Assay, samples were allowed to enrich for a total of 24 ± 2 h at 37 ± 1°C. Enriched samples were mixed thoroughly before taking aliquots for analysis. Fifty microliters were streaked onto Oxoid Brilliance *Cronobacter sakazakii* agar and incubated for 24 ± 2 h at 37 ± 1°C. The presence of colonies that grew well (1–3 mm) and appeared blue-green indicated a positive sample. For confirmation per FDA BAM Chapter 29, sections E and F were performed (1). Briefly, from a 24 h enrichment, 2 × 40 mL aliquots were centrifuged at 3000 × *g* for 10 min. The supernatant was discarded and the resultant pellets were resuspended in 200 µL sterile phosphate buffered saline. One hundred microliters aliquots of the resuspended pellet were plated on two Druggan-Forsythe-Iversen (DFI) chromogenic agar and two R&F^®^*Cronobacter* chromogenic agar plates. In addition, a loopful of each enrichment was streaked onto two DFI chromogenic agar and two R&F^®^*Cronobacter* chromogenic agar plates. All plates were incubated at 36 ± 1°C for 18–24 h. Presumptive positive colonies were confirmed by PCR as outlined in section F of FDA BAM Chapter 29 (1).

##### ISO 22964:2006

ISO 22964:2006, the current version at time of testing, was used in the method developer laboratory for the matrix evaluation. Briefly, 90 mL of BPW was added to 10 g PIF. The samples were incubated at 37 ± 1°C for 18 ± 2 h. Then 0.1 mL was transferred from the BPW culture to 10 mL modified lauryl sulphate broth (mLST)/vancomycin medium and incubated at 44 ± 1°C for 24 ± 2 h. A loopful of mLST/vancomycin culture was streaked onto *Enterobacter sakazakii* Isolation Agar and incubated at 44 ± 1°C for 24 ± 2 h. One to five presumptive positive colonies were then streaked onto tryptic soya agar (TSA) plates and incubated at 25°C for 48 ± 4 h. Yellow pigmented colonies were chosen for further biochemical confirmation tests (2).

##### FDA BAM Chapter 29

For the FDA BAM Chapter 29 *Cronobacter* method, 900 mL of sterile BPW was added to 100 g PIF in sterile 2 L Erlenmeyer flasks and gently agitated by hand until PIF was uniformly suspended. Test samples were incubated at 36 ± 1°C for 24 ± 2 h. After enrichment, the samples were thoroughly mixed and 4 × 40 mL from each sample were transferred into 50 mL centrifuge tubes. The aliquots were centrifuged at 3000 × *g* for 10 min and the supernatant was discarded. The resultant pellet was resuspended in 200 µL phosphate buffered saline. Two aliquots were used for PCR to determine presumptive positives and two aliquots were used for cultural confirmation if necessary. For the PCR screen, two aliquots were transferred to 1.5 mL microcentrifuge tubes and centrifuged at 3000 × *g* for 5 min. The supernatant was discarded and the pellet was resuspended in 400 µL PrepMan Ultra^®^ sample preparation reagent and mixed by vortex at maximum speed until the pellet was completely resuspended. The samples were heated in a dry bath incubator at 100°C for 10 min, then cooled to room temperature. Once the samples reached room temperature, the samples were centrifuged for 2 min at 15 000 × *g* and a 50 µL aliquot of the supernatant was transferred to a new microcentrifuge tube for PCR analysis. For each sample, PCR analyses were performed with and without internal control (InC). The PCR reaction components and the PCR protocol was followed as outlined in the FDA BAM Chapter 29 reference method. Presumptive positives were confirmed using FDA BAM Chapter 29, sections E and F. Briefly, 100 µL aliquots of the resuspended pellet were plated on two DFI chromogenic agar and two R&F^®^*Cronobacter* chromogenic agar plates. In addition, a loopful of each enrichment was streaked onto two DFI chromogenic agar and two R&F^®^*Cronobacter* chromogenic agar plates. All plates were incubated at 36 ± 1°C for 18–24 h. Colonies were confirmed by PCR as outlined in section F of BAM Chapter 29 (1).

**Table 5.** PhageDx *Cronobacter* Assay versus ISO 22964 method comparison results

Matrix^a^	Strain	MPN/test portion^b^	*n* ^c^	PhageDx *Cronobacter* result			ISO 22964			dPOD_CP_^g^	95% CI^h^
				x^d^	POD_CP_^e^	95% CI	x	POD_CC_^f^	95% CI		
PIF (10 g, milk-based)	*C. muytjensii* FSL-F6-031	N/A^i^	5	0	0.00	0.00, 0.43	0	0.00	0.00, 0.43	0.00	−0.47, 0.47
		0.46 (0.26, 0.72)	20	6	0.30	0.15, 0.52	6	0.30	0.15, 0.52	0.00	−0.13, 0.13
		1.74 (0.77, 4.03)	5	4	0.80	0.38, 1.00	4	0.80	0.38, 1.00	0.00	−0.47, 0.47
PIF (10 g, soy-based)	*C. malonaticus* ES686	N/A	5	0	0.00	0.00, 0.43	0	0.00	0.00, 0.43	0.00	−0.47, 0.47
		0.78 (0.46, 1.27)	20	10	0.50	0.30, 0.70	10	0.50	0.30, 0.70	0.00	−0.13, 0.13
		4.03 (2.14, 11.5)	5	5	1.00	0.57, 1.00	5	1.00	0.57, 1.00	0.00	−0.47, 0.47
PIF (10 g, milk- based)^j^	*C. sakazakii* ATCC 29544	N/A	5	0	0.00	0.00, 0.43	0	0.00	0.00, 0.43	0.00	−0.47, 0.47
		0.49 (0.25, 0.85)	20	7	0.35	0.18, 0.57	7	0.35	0.18, 0.57	0.00	−0.13, 0.13
		1.61 (0.75, 3.44)	5	5	1.00	0.57, 1.00	5	1.00	0.57, 1.00	0.00	−0.47, 0.47

a Matrix study is paired.

b MPN is based on the POD of reference method test portions using the Least Cost Formulations MPN calculator, with 95% confidence interval.

c *n* = Number of test portions.

d x = Number of positive test portions.

e POD_CP_ = Candidate method presumptive positive outcomes confirmed positive.

f POD_CC_ = Reference method confirmed positive outcomes divided by the total number of trials.

g dPOD_CP_ = Difference between the candidate method and reference method POD values.

h 95% CI = If the confidence interval of a dPOD does not contain zero, then the difference is statistically significant at the 5% level.

i N/A = Not applicable.

j Matrix tested by the independent laboratory.

**Table 6.** PhageDx *Cronobacter* Assay presumptive versus confirmed (per FDA BAM Chapter 29) results—POD result

Matrix	Strain	MPN/test portion^a^	*n* ^b^	PhageDx presumptive result			PhageDx confirmed result			dPOD_CP_^f^	95% CI^g^
				x^c^	POD_CP_^d^	95% CI	x	POD_CC_^e^	95% CI		
PIF (100 g, milk-based)	*C. muytjensii* FSL-F6-031	N/A^h^	5	0	0.00	0.00, 0.43	0	0.00	0.00, 0.43	0.00	−0.47, 0.47
		0.32 (0.13, 0.56)	20	12	0.60	0.39, 0.78	12	0.60	0.39, 0.78	0.00	−0.13, 0.13
		3.04 (1.50, 6.17)	5	4	0.80	0.38, 1.00	4	0.80	0.38, 1.00	0.00	−0.47, 0.47
PIF (300 g, milk-based)	*C. muytjensii* FSL-F6-031	N/A	5	0	0.00	0.00, 0.43	0	0.00	0.00, 0.43	0.00	−0.47, 0.47
		0.32 (0.13, 0.56)	20	11	0.55	0.34, 0.74	11	0.55	0.34, 0.74	0.00	−0.13, 0.13
		3.04 (1.50, 6.17)	5	5	1.00	0.57, 1.00	5	1.00	0.57, 1.00	0.00	−0.47, 0.47
PIF (100 g, soy-based)	*C. malonaticus* ES686	N/A	5	0	0.00	0.00, 0.43	0	0.00	0.00, 0.43	0.00	−0.47, 0.47
		0.70 (0.40, 1.34)	20	9	0.45	0.26, 0.66	9	0.45	0.26, 0.66	0.00	−0.13, 0.13
		2.40 (1.19, 4.86)	5	5	1.00	0.57, 1.00	5	1.00	0.57, 1.00	0.00	−0.47, 0.47
PIF (300 g, soy-based)	*C. malonaticus* ES686	N/A	5	0	0.00	0.00, 0.43	0	0.00	0.00, 0.43	0.00	−0.47, 0.47
		0.70 (0.40, 1.34)	20	9	0.45	0.26, 0.66	9	0.45	0.26, 0.66	0.00	−0.13, 0.13
		2.40 (1.19, 4.86)	5	5	1.00	0.57, 1.00	5	1.00	0.57, 1.00	0.00	−0.47, 0.47
PIF (100 g, milk-based)^i^	*C. sakazakii* ATCC 29544	N/A	5	0	0.00	0.00, 0.43	0	0.00	0.00, 0.43	0.00	−0.47, 0.47
		1.05 (0.64, 1.71)	20	12	0.60	0.39, 0.78	12	0.60	0.39, 0.78	0.00	−0.13, 0.13
		2.28 (1.11, 4.70)	5	5	1.00	0.57, 1.00	5	1.00	0.57, 1.00	0.00	−0.47, 0.47
PIF (300 g, milk-based)^i^	*C. sakazakii* ATCC 29544	N/A	5	0	0.00	0.00, 0.43	0	0.00	0.00, 0.43	0.00	−0.47, 0.47
		1.05 (0.64, 1.71)	20	14	0.70	0.48, 0.85	14	0.70	0.48, 0.85	0.00	−0.13, 0.13
		2.28 (1.11, 4.70)	5	5	1.00	0.57, 1.00	5	1.00	0.57, 1.00	0.00	−0.47, 0.47

a MPN is based on the POD of reference method test portions using the Least Cost Formulations MPN calculator, with 95% confidence interval.

b *n* = Number of test portions.

c x = Number of positive test portions.

d POD_CP_ = Candidate method presumptive positive outcomes divided by the total number of trials.

e POD_CC_ = Candidate method confirmed positive (per FDA BAM Chapter 29) outcomes divided by the total number of trials.

f dPOD_CP_ = Difference between the candidate method presumptive result and candidate method confirmed result POD values.

g 95% CI = If the confidence interval of a dPOD does not contain zero, then the difference is statistically significant at the 5% level.

h N/A = Not applicable.

i Matrix tested by the independent laboratory.

**Table 7.** PhageDx *Cronobacter* Assay presumptive versus confirmed (per PhageDx confirmation procedure) results—POD result

Matrix	Strain	MPN/test portion^a^	*n* ^b^	PhageDx presumptive result			PhageDx confirmed result			dPOD_CP_^f^	95% CI^g^
				x^c^	POD_CP_^d^	95% CI	x	POD_CC_^e^	95% CI		
PIF (100 g, milk-based)	*C. muytjensii* FSL-F6-031	N/A^h^	5	0	0.00	0.00, 0.43	0	0.00	0.00, 0.43	0.00	−0.47, 0.47
		0.32 (0.13, 0.56)	20	12	0.60	0.39, 0.78	12	0.60	0.39, 0.78	0.00	−0.13, 0.13
		3.04 (1.50, 6.17)	5	4	0.80	0.38, 1.00	4	0.80	0.38, 1.00	0.00	−0.47, 0.47
PIF (300 g, milk-based)	*C. muytjensii* FSL-F6-031	N/A	5	0	0.00	0.00, 0.43	0	0.00	0.00, 0.43	0.00	−0.47, 0.47
		0.32 (0.13, 0.56)	20	11	0.55	0.34, 0.74	11	0.55	0.34, 0.74	0.00	−0.13, 0.13
		3.04 (1.50, 6.17)	5	5	1.00	0.57, 1.00	5	1.00	0.57, 1.00	0.00	−0.47, 0.47
PIF (100 g, soy-based)	*C. malonaticus* ES686	N/A	5	0	0.00	0.00, 0.43	0	0.00	0.00, 0.43	0.00	−0.47, 0.47
		0.70 (0.40, 1.34)	20	9	0.45	0.26, 0.66	9	0.45	0.26, 0.66	0.00	−0.13, 0.13
		2.40 (1.19, 4.86)	5	5	1.00	0.57, 1.00	5	1.00	0.57, 1.00	0.00	−0.47, 0.47
PIF (300 g, soy-based)	*C. malonaticus* ES686	N/A	5	0	0.00	0.00, 0.43	0	0.00	0.00, 0.43	0.00	−0.47, 0.47
		0.70 (0.40, 1.34)	20	9	0.45	0.26, 0.66	9	0.45	0.26, 0.66	0.00	−0.13, 0.13
		2.40 (1.19, 4.86)	5	5	1.00	0.57, 1.00	5	1.00	0.57, 1.00	0.00	−0.47, 0.47
PIF (100 g, milk-based)^i^	*C. sakazakii* ATCC 29544	N/A	5	0	0.00	0.00, 0.43	0	0.00	0.00, 0.43	0.00	−0.47, 0.47
		1.05 (0.64, 1.71)	20	12	0.60	0.39, 0.78	12	0.60	0.39, 0.78	0.00	−0.13, 0.13
		2.28 (1.11, 4.70)	5	5	1.00	0.57, 1.00	5	1.00	0.57, 1.00	0.00	−0.47, 0.47
PIF (300 g, milk-based)^i^	*C. sakazakii* ATCC 29544	N/A	5	0	0.00	0.00, 0.43	0	0.00	0.00, 0.43	0.00	−0.47, 0.47
		1.05 (0.64, 1.71)	20	14	0.70	0.48, 0.85	14	0.70	0.48, 0.85	0.00	−0.13, 0.13
		2.28 (1.11, 4.70)	5	5	1.00	0.57, 1.00	5	1.00	0.57, 1.00	0.00	−0.47, 0.47

a MPN is based on the POD of reference method test portions using the Least Cost Formulations MPN calculator, with 95% confidence interval.

b *n* = Number of test portions..

c x = Number of positive test portions.

d POD_CP_ = Candidate method presumptive positive outcomes divided by the total number of trials.

e POD_CC_ = Candidate method confirmed positive (per PhageDx confirmation procedure) outcomes divided by the total number of trials.

f dPOD_CP_ = Difference between the candidate method presumptive result and candidate method confirmed result POD values.

g 95% CI = If the confidence interval of a dPOD does not contain zero, then the difference is statistically significant at the 5% level.

h N/A = Not applicable.

i Matrix tested by the independent laboratory.

**Table 8.** PhageDx *Cronobacter* Assay versus FDA BAM Chapter 29 method comparison results—POD result

Matrix^a^	Strain	MPN/test portion^b^	*n* ^c^	PhageDx *Cronobacter* result			FDA BAM Ch. 29			dPOD_C_^g^	95% CI^h^
				x^d^	POD_C_^e^	95% CI	x	POD_R_^f^	95% CI		
PIF (100 g, milk-based)	*C. muytjensii* FSL-F6-031	N/A^i^	5	0	0.00	0.00, 0.43	0	0.00	0.00, 0.43	0.00	−0.43, 0.43
		0.32 (0.13, 0.56)	20	12	0.60	0.39, 0.78	5	0.25	0.11, 0.47	0.35	0.04, 0.58^j^
		3.04 (1.50, 6.17)	5	4	0.80	0.38, 1.00	5	1.00	0.57, 1.00	−0.20	−0.62, 0.28
PIF (300 g, milk-based)	*C. muytjensii* FSL-F6-031	N/A	5	0	0.00	0.00, 0.43	0	0.00	0.00, 0.43	0.00	−0.43, 0.43
		0.32 (0.13, 0.56)	20	11	0.55	0.34, 0.74	5	0.25	0.11, 0.47	0.30	0.00, 0.54
		3.04 (1.50, 6.17)	5	5	1.00	0.57, 1.00	5	1.00	0.57, 1.00	0.00	−0.43, 0.43
PIF (100 g, soy-based)	*C. malonaticus* ES686	N/A	5	0	0.00	0.00, 0.43	0	0.00	0.00, 0.43	0.00	−0.43, 0.43
		0.70 (0.40, 1.34)	20	9	0.45	0.26, 0.66	11	0.55	0.34, 0.75	−0.10	−0.37, 0.19
		2.40 (1.19, 4.86)	5	5	1.00	0.57, 0.66	5	1.00	0.57, 1.00	0.00	−0.43, 0.43
PIF (300 g, soy-based)	*C. malonaticus* ES686	N/A	5	0	0.00	0.00, 0.43	0	0.00	0.00, 0.43	0.00	−0.43, 0.43
		0.70 (0.40, 1.34)	20	9	0.45	0.26, 0.66	11	0.55	0.34, 0.75	−0.10	−0.37, 0.19
		2.40 (1.19, 4.86)	5	5	1.00	0.57, 0.66	5	1.00	0.57, 1.00	0.00	−0.43, 0.43
PIF (100 g, milk-based)^k^	*C. sakazakii* ATCC 29544	N/A	5	0	0.00	0.00, 0.43	0	0.00	0.00, 0.43	0.00	−0.43, 0.43
		1.05 (0.64, 1.71)	20	12	0.60	0.39, 0.78	12	0.55	0.39, 0.78	0.00	−0.28, 0.28
		2.28 (1.11, 4.70)	5	5	1.00	0.57, 1.00	5	1.00	0.57, 1.00	0.00	−0.43, 0.43
PIF (300 g, milk-based)^k^	*C. sakazakii* ATCC 29544	N/A	5	0	0.00	0.00, 0.43	0	0.00	0.00, 0.43	0.00	−0.43, 0.43
		1.05 (0.64, 1.71)	20	14	0.70	0.48, 0.85	12	0.60	0.39, 0.78	0.10	−0.18, 0.36
		2.28 (1.11, 4.70)	5	5	1.00	0.57, 1.00	5	1.00	0.57, 1.00	0.00	−0.43, 0.43

a Matrix test portion for the PhageDx *Cronobacter* method is listed. Portions were compared to FDA BAM Chapter 29 100 g test portions.

b MPN is based on the POD of reference method test portions using the Least Cost Formulations MPN calculator, with 95% confidence interval.

c *n* = Number of test portions.

d x = Number of positive test portions.

e POD_C_ = Candidate method presumptive positive outcomes confirmed positive were identical using both confirmation procedures, hence one result is reported here.

f POD_R_ = Reference method confirmed positive outcomes divided by the total number of trials.

g dPOD_C_ = Difference between the candidate method and reference method POD values.

h 95% CI = If the confidence interval of a dPOD does not contain zero, then the difference is statistically significant at the 5% level.

i N/A = Not applicable.

j Difference in number of positive samples between methods in unpaired test contributed to statistical difference. No false positives or false negatives were observed.

k Matrix tested by the independent laboratory.

All test results were analyzed using *POD* statistical analysis to 95% confidence intervals (*CI*). *POD* analysis is described in the AOAC INTERNATIONAL guidelines in Appendix J (9). Data from the analysis are presented in Tables 5–8.

### Independent Laboratory Validation Study

The independent laboratory evaluation included a matrix study for milk-based PIF comparing the PhageDx *Cronobacter* Assay to ISO 22964:2017 and FDA BAM Chapter 29 reference methods (1, 3). For the method comparison to ISO 22964:2017, 30 paired 10 g test portions were evaluated. For the method comparison to FDA BAM Chapter 29, 100 g and 300 g test portions of the PhageDx *Cronobacter* Assay were compared to 100 g test portions of the reference method. Within each sample set, there were five uninoculated samples (0 CFU/test portion), 20 low level inoculated samples (0.2–2 CFU/test portion), and five high level inoculated samples (2–10 CFU/test portion). The low inoculation level was designed to produce fractional positive results, those in which the candidate or reference method produced 5–15 positive results (25–75%).

The PIF was purchased from a local distributor, prescreened for natural contamination of the analyte following ISO 22964:2017, and analyzed for total aerobic count by FDA BAM Chapter 3. Following the screening, the matrix was inoculated with a strain of *Cronobacter* species. For the validation, a lyophilized culture was used to inoculate the PIF. The lyophilized culture was prepared by transferring a single *C. sakazakii* colony from TSA with 5% sheep blood into brain heart infusion (BHI) broth and incubating the culture at 35 ± 2°C for 18–24 h. Following incubation, the culture was diluted in a sterile cryoprotectant, reconstituted nonfat dry milk (NFDM), and placed onto a freeze dry system for 48–72 h. After removing the culture from the freeze dry system, the lyophilized culture was diluted in NFDM to a low level expected to yield fractional positive results and a high level expected to yield all positive results. A bulk lot of the matrix was inoculated. After inoculation, the matrix was held for 2 weeks at room temperature (24 ± 2°C) to allow for equilibration of the organism in the matrix.

Total aerobic count was determined according to FDA BAM Chapter 3. The level of *Cronobacter* in the low level inoculum and high level inoculum was determined by MPN on the day of analysis. For the paired sample analysis, the low level MPN was determined by evaluating 5 × 25 g test portions, the 20 × 10 g test portions from the study, and 5 × 4 g test portions. The level of *Cronobacter* in the high level inoculum was determined by evaluating the 5 × 10 g test portions from the study, 5 × 4 g test portions, and 5 × 1.5 g test portions.

For the unpaired analysis, the low level MPN was determined by evaluating 5 × 200 g test portions, the 20 × 100 g reference method test portions from the study, and 5 × 50 g test portions. The level of *Cronobacter* in the high level inoculum was determined by evaluating the 5 × 100 g reference method test portions from the study, 5 × 50 g test portions, and 5 × 25 g test portions. Each test portion was enriched with BPW and analyzed by the reference method procedure. The number of positives from the three test levels was used to calculate the MPN using the LCF MPN calculator (version 1.6) (11).

####  

##### ISO 22964:2017

For ISO 22964:2017, 10 g PIF test portions were enriched with 90 mL BPW (ISO formulation) and incubated at 37 ± 1°C for 18 ± 2 h. Following incubation, 0.1 mL of primary enrichment was transferred into 10 mL *Cronobacter* selective broth (CSB) and incubated at 41.5 ± 1°C for 24 ± 2 h. Following incubation, a loopful of the CSB was streaked to chromogenic *Cronobacter* isolation (CCI) agar and incubated at 41.5 ± 1°C for 24 ± 2 h. Following incubation of the CCI plates, one to five typical *Cronobacter* species colonies (medium sized colonies, 1–3 mm, blue-green to blue) were transferred to TSA and incubated at 35 ± 1°C for 18 to 24 h. After incubation, an oxidase test was conducted on a typical colony (yellow-pigmented, 1–3 mm) and final biochemical confirmation was performed by using the VITEK^®^ 2 GN Biochemical Identification card following AOAC *Official Method* **2011.17** (12).

##### FDA BAM Chapter 29

For FDA BAM Chapter 29, 100 g PIF test portions were added to 2 L Erlenmeyer flasks, enriched with 900 mL pre-warmed (37°C) BPW, and incubated at 37 ± 1°C for 24 ± 2 h. Following incubation, 4 × 40 mL aliquots were transferred to 4 × 50 mL conical vials. The aliquots were centrifuged at 3000 × *g* for 10 min. For each conical tube, the supernatants were aspirated and the lipid precipitate was removed using sterile cotton swabs. The remaining pellet was re-suspended by adding 200 µL phosphate buffered saline and mixing the suspension by vortex at max speed for 20 s. For each sample, two of the aliquots were used for PCR screening of *Cronobacter* and two of the aliquots were used for cultural confirmation.

For the PCR screening, two aliquots were transferred to separate 1.5 mL microcentrifuge tubes and centrifuged at 3000 × *g* for 5 min. The supernatant and lipid layer were removed and the pellet was re-suspended by adding 400 µL PrepMan Ultra sample preparation reagent and mixing by vortex at max speed until suspension was achieved. The samples were heat treated in a dry bath incubator at 100°C for 10 min, then cooled to room temperature. Once the samples reached room temperature, the samples were centrifuged for 2 min at 15 000 × *g* and a 50 µL aliquot of the supernatant was transferred to a new microcentrifuge tube for PCR analysis. For each sample, PCR analyses were performed with and without InC. The PCR reaction components and PCR protocol were followed as outlined in the FDA BAM Chapter 29 reference method.

Regardless of the presumptive PCR result, a 100 µL aliquot of suspended cells from each sample was streaked onto two DFI chromogenic agar plates and two R&F^®^ agar plates. DFI chromogenic agar plates and R&F^®^ agar plates were incubated at 36 ± 1°C for 18–24 h. Following incubation, typical *Cronobacter* colonies from DFI chromogenic agar (weak to dark green, brownish colonies, or green-centered colonies with a white to yellow border) and R&F^®^ agar plates (blue to black or blue to grey colonies with a red background) were biochemically confirmed by VITEK 2 GN Biochemical Identification card (AOAC *Official Method* **2011.17**) and PCR analysis (12).

## Results

Inclusivity and exclusivity studies show that the PhageDx *Cronobacter* Assay is specific for *Cronobacter* spp. The PhageDx *Cronobacter* Assay demonstrates 100% inclusivity with the 75 *Cronobacter* strains tested (Table 1). The PhageDx *Cronobacter* Assay also demonstrates exclusivity for 35/38 non-*Cronobacter* strains tested (Table 2). The three non-*Cronobacter* strains which were detected by the PhageDx *Cronobacter* Assay were from the closely related *Enterobacter* genus. That *Cronobacter* was formerly named *Enterobacter* indicates how closely related these two genera are, thus it is not entirely surprising that there may be some cross-reactivity with selected members of this family.

Product consistency and stability studies demonstrate that the PhageDx *Cronobacter* recombinant phages can be manufactured consistently and are stable for at least 3 months when stored at 4°C. Working solutions of each lot produced similar results when tested according to QC tests for bacteriophage concentration, background signal, and LOD. Stability tests of each lot were performed to determine the shelf life of the recombinant phage. These tests demonstrated that lots produced 1 month prior to testing showed no significant difference from lots produced 3 months prior to testing. Additionally, no variation in exclusivity was observed with these three recombinant phage lots in tests with *C. koseri* (Table 3).

Robustness testing of the PhageDx *Cronobacter* Assay demonstrated that variations in enrichment time, recombinant phage concentration, and luciferase substrate working solution amount do not alter the results compared to the standard protocol. Enrichment times of 14 and 24 h, recombinant phage volumes of 8 and 12 µL, and luciferase substrate working solution volumes of 45 and 55 µL produced identical results to the standard protocol of 16 h enrichment, 10 µL recombinant phage, and 50 µL luciferase substrate working solution in both uninoculated and low inoculum test samples (Table 4). These results indicate that these deviations from the PhageDx *Cronobacter* Assay protocol did not alter the final results.

The method developer matrix studies showed that there were no differences between the PhageDx *Cronobacter* Assay and the ISO 22964:2006 and the FDA BAM Ch. 29 *Cronobacter* reference methods for all matrixes tested (Tables 5–8). All test portions that were presumptive positives by PhageDx *Cronobacter* Assay were confirmed by their respective reference methods to contain *Cronobacter*. There were no false negative results. The POD analyses indicated no significant differences exist between the PhageDx *Cronobacter* Assay and the ISO 22964:2006 reference method in a paired study (Table 5). There was no statistical difference between the number of PhageDx *Cronobacter* Assay presumptive positive results and the FDA BAM Chapter 29 confirmation results (Table 6). Likewise, comparison of the PhageDx *Cronobacter* Assay presumptive results to either the FDA BAM Chapter 29 confirmation or the Oxoid Brilliance *Cronobacter* sakazakii agar plating confirmation were not significantly different (Tables 6 and 7). The comparison of the PhageDx *Cronobacter* Assay and the FDA BAM Chapter 29 unpaired study showed that the there was no statistical difference in the performance of the two methods (Table 8). The one exception was the 100 g milk-based PhageDx Assay versus FDA BAM Ch. 29 method comparison (Table 8). The difference between the fractional positives was statistically significant, where the dPOD was 0.35, and the CI was (0.04, 0.58). The aerobic plate count of the PIF used in the study was 0 CFU/g, indicating that the PIF had no or very low levels of background flora present at the initiation of the enrichment process.

Matrix studies done by an independent laboratory further support the claim that the performance of the PhageDx *Cronobacter* Assay is equivalent to that of FDA BAM Ch. 29 and and ISO 22964 reference methods. For all three levels, the POD analyses between the PhageDx *Cronobacter* Assay and the reference methods indicated that there was no statistically significant difference at the 5% level between the number of positive results obtained by the methods (Tables 5–8). For all three levels, the POD analyses between presumptive results of the PhageDx *Cronobacter* Assay and confirmed results indicated that there was no statistically significant difference at the 5% level for all test portions analyzed (Tables 5–8). The aerobic plate count of the PIF used in the study was 40 CFU/g, indicating that the PIF had approximately 400 CFU (10 g), 4000 CFU (100 g), or 12 000 CFU (300 g) of background flora present at the initiation of the enrichment process.

## Discussion

The results of this validation study show that the PhageDx *Cronobacter* Assay is an effective alternative to the ISO 22964:2006/2017 for the detection of *Cronobacter* in 10 g of milk- and soy-based PIF and FDA BAM Chapter 29 for the detection of *Cronobacter* in 100 g or 300 g of milk- and soy-based PIF. In inclusivity and exclusivity testing, the method was shown to be specific for *Cronobacter*, correctly identifying all 75 *Cronobacter* target strains and 35 non-target strains. The PhageDx *Cronobacter* Assay displayed cross reactivity with some closely related strains of *Enterobacter*. *Cronobacter* was formerly categorized in the genus *Enterobacter*. This indicates how closely related these two genera are, thus it is not entirely surprising that there may be some cross-reactivity with selected members of this family.

The recombinant phage can be produced consistently and is stable for 3 months when stored appropriately. Robustness testing of the PhageDx *Cronobacter* Assay indicated that the method works well when the assay parameters (enrichment time, recombinant phage concentration, and substrate amount) were varied from the stated protocol. Method developer studies demonstrated that the performance of the PhageDx *Cronobacter* Assay was not statistically different from that of ISO 22964 for 10 g test sample or FDA BAM Chapter 29 for 100 g and 300 g test samples. One exception was the comparison of the PhageDx Assay and FDA BAM Chapter 29 for the 100 g milk-based fractional positives data which was statistically significant (Table 8). One possible explanation is that this could be a result of skewed sample inoculation (PhageDx = 12, FDA BAM = 5). Alternatively, the PhageDx *Cronobacter* Assay may be more sensitive and was able to detect a greater number of presumptive positives than the FDA BAM Chapter 29 presumptive positive PCR method. However, since no false positives or false negatives were found in the study, it suggests that this result is likely a product of one or more of these factors. Independent laboratory testing demonstrated that the PhageDx *Cronobacter* Assay was able to detect *Cronobacter* at low levels in 10, 100, and 300 g PIF, which also contained approximately 40 CFU/g background flora, and an alternative confirmation procedure was shown to be identical to the reference method confirmation procedures.

The PhageDx *Cronobacter* Assay also has a number of advantages over the ISO 22964 and FDA BAM Chapter 29 reference methods. In addition to being a specific assay, the results are easy to interpret as an RLU end point is used to determine the outcome of the assay. This is in contrast to the ISO method where interpretation of reagent color changes is required or the FDA BAM method where PCR amplification plots may have to be assessed. With the PhageDx *Cronobacter* Assay, test samples with an RLU of 500 or greater are considered positive. Another advantage is that PhageDx provides a presumptive positive result in as little as 18.5 h compared to >24 h in the case of FDA BAM and >60 h in the case of ISO method. PhageDx is also a simple test that involves only five basic steps: enrichment, dilution, infection, substrate addition, and signal readout. Finally, the PhageDx *Cronobacter* Assay is a rapid method that offers considerable cost and time savings compared to the ISO 22964 and FDA BAM Chapter 29 reference methods.

## Conclusion

Results of this validation study support the claim that the PhageDx *Cronobacter* Assay is a specific, sensitive, fast, and simple method for the detection of *Cronobacter* in PIF and is statistically comparable to the ISO:22964:2006/2017 and FDA BAM Chapter 29 *Cronobacter* methods. By using a luciferase-expressing recombinant bacteriophage, the assay was able to detect a single, viable bacterium after a 16 h enrichment and a 2 h infection. The PhageDx *Cronobacter* Assay thus offers shorter time to results compared with the other validated *Cronobacter* detection assays. The PhageDx *Cronobacter* Assay provides PIF manufacturers with an alternative method for conducting required regulatory testing that is easier to use and potentially more cost effective than current validated methods for *Cronobacter* detection.
